# Emerging Roles of Fibroblast Growth Factor 10 in Cancer

**DOI:** 10.3389/fgene.2018.00499

**Published:** 2018-10-24

**Authors:** Natasha S. Clayton, Richard P. Grose

**Affiliations:** Centre for Tumour Biology, Barts Cancer Institute, CRUK Centre of Excellence, Queen Mary University of London, London, United Kingdom

**Keywords:** FGF10, FGFR2, FGFR2b, FGFR1, FGFR1b, cancer

## Abstract

Whilst cross-talk between stroma and epithelium is critical for tissue development and homeostasis, aberrant paracrine stimulation can result in neoplastic transformation. Chronic stimulation of epithelial cells with paracrine Fibroblast Growth Factor 10 (FGF10) has been implicated in multiple cancers, including breast, prostate and pancreatic ductal adenocarcinoma. Here, we examine the mechanisms underlying FGF10-induced tumourigenesis and explore novel approaches to target FGF10 signaling in cancer.

## Introduction

The majority of secreted FGFs signal in an autocrine or paracrine manner, by binding to FGF receptors (FGFR1-4) on the surface of target cells. Alternative splicing of the immunoglobin-like domain III in FGFR1-FGFR3 produces two variants, IIIb and IIIc, which confer different ligand binding specificities. FGF10, a member of the FGF7 subfamily, signals in a paracrine manner through activation of the IIIb splice variants of FGFR2 (FGFR2b) and FGFR1 (FGFR1b), which are predominantly expressed on the surface of cells of epithelial origin. Whilst *in vitro* assays have shown FGF10 to activate FGFR1b more weakly than FGFR2b, FGFR1b activation *in vivo* may be achieved in scenarios where extracellular FGF10 reaches high concentrations (Zhang et al., 2006; Ornitz and Itoh, 2015). It has been suggested that FGF10 may also perform intracrine roles within FGF10-producing cells, primarily through trafficking to the nucleus. Whilst the functional significance of FGF10 nuclear localization remains to be determined, disrupted nuclear localization of FGF10 has been linked to lacrimo-auriculo-denot-digital syndrome (Mikolajczak et al., 2016).

FGF10 is known to be critical for brain, lung and limb development (Sekine et al., 1999; Hajihosseini et al., 2008) and contributes to wound healing and tissue repair by promoting cell migration and proliferation (Werner et al., 1994; Volckaert et al., 2011). Given this role of FGF10 in adult tissues, it is unsurprising that aberrant signaling of FGF10 through FGFR2b, and in some instances FGFR1b, contributes to the progression of a number of human cancers.

## FGF10 in Breast Cancer

**FIGURE 1:**
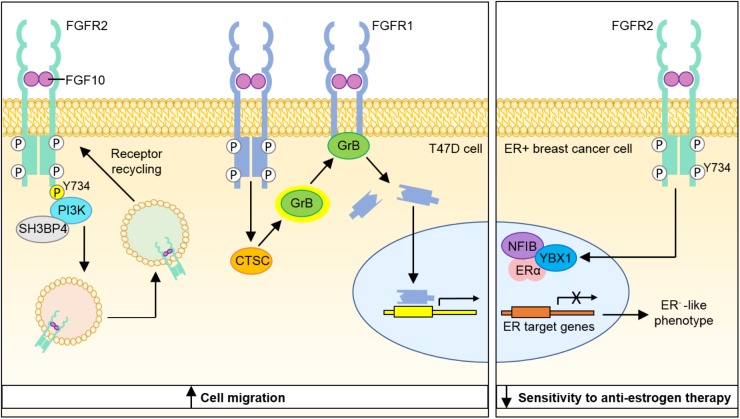
Model depicting molecular mechanisms through which FGF10-FGFR1 and FGF10-FGFR2 signaling may contribute to breast cancer progression. Binding of FGF10 to FGFR2b leads to phosphorylation of the receptor at Y734 and recruitment of PI3K and SH3BP4, which promote receptor recycling and increased cell migration. FGF10 binding to FGFR1 leads to cleavage of the receptor by granzyme B and the translocation of a 55 kDa fragment of FGFR1 to the nucleus, leading to increased cell migration. Stimulation of ER^+^ breast cancer cells with FGF10 enhances the interaction of NFIB and YBX1 with the ER and inhibits its transcriptional activity to produce a more ER^−^ phenotype with lower sensitivity to anti-estrogen therapy. PI3K, phosphatidylinositide 3-kinase; SH3BP4, SH3-binding protein 4; CTSC, cathepsin C; GrB, granzyme B; NFIB, nuclear factor I B; YBX1, Y-Box Binding Protein-1; ERα, estrogen receptor α.

Studies in Fgf10^−/−^ and Fgfr2b^−/−^ mouse embryos demonstrate that FGF10-FGFR2b signaling plays a key role in mammary gland development (Mailleux et al., 2002; Veltmaat et al., 2006). Whilst FGF10 is not expressed in the luminal epithelial cells of the normal human breast duct (Grigoriadis et al., 2006), transcription of the *FGF10* gene is elevated 10% of breast carcinomas (Theodorou et al., 2004) and genome-wide association studies have identified variants near the *FGF10* locus as a genetic risk factor for breast cancer susceptibility (Stacey et al., 2008). Similarly, SNPs affecting FGFR2b expression have been correlated with breast cancer susceptibility (Meyer et al., 2008; Fachal and Dunning, 2015) and amplification of *FGFR1*, occurring in up to 12% of breast cancer cases (Cerami et al., 2012; Gao et al., 2013; Pereira et al., 2016), is correlated with poor prognosis (Reis-Filho et al., 2006; Elbauomy Elsheikh et al., 2007). A number of *in vitro* studies have shed light on the cellular roles of FGF10-FGFR2/1 signaling in breast cancer cell behavior (Figure 1).

FGFR2 activation has been shown to repress the activity of the estrogen receptor (ER) regulon (Campbell T. M. et al., 2016), which has been correlated with poor prognosis in a cohort of ER^+^ breast cancer patients (Castro et al., 2016). Stimulation of the ER^+^ breast cancer cell line MCF-7 with FGF10 enhanced the interaction of the transcription factors NFIB and YBX1 with the ER, which inhibited its transcriptional activity and shunted the cells toward a more ER^−^, basal-like cancer phenotype with reduced estrogen dependency and lower sensitivity to anti-estrogen therapy. Treatment of ER^+^ breast cancer cell lines with the FGFR inhibitors AZD4547 and PD173074 sensitized the cells to the anti-estrogen tamoxifen, suggesting that targeting FGF10-FGFR2 signaling may offer a new approach to overcoming resistance to hormone-deprivation therapy in ER^+^ breast cancer (Campbell et al., 2018).

Nuclear localization of receptor tyrosine kinases (RTKs) has been documented for 12 RTK families (Chen and Hung, 2015) and has been correlated with poor prognosis in various cancers (Aleksic et al., 2010; Hadžisejdić et al., 2010; Traynor et al., 2013; Coleman et al., 2014). *In vitro* studies using breast cancer cell lines showed that FGF10 stimulation lead to the nuclear translocation of a 55 kDa C-terminal fragment of FGFR1, which in turn promoted the transcription of genes that stimulate cell migration and invasion. Cleavage of FGFR1 to yield this C-terminal fragment was found to be mediated by granzyme B activity, which was itself positively regulated by FGF10 stimulation. In 3D organotypic cell culture models, FGFR1 nuclear localization was most apparent in invading cells. Importantly, increased nuclear FGFR1 staining was also detected in tissue sections of invasive breast carcinoma (Chioni and Grose, 2012).

Analysis of FGFR2b signaling networks *in vitro* revealed that stimulation of FGFR2b with FGF10 promoted receptor recycling and led to an increase in breast cancer migration, whilst stimulation of FGFR2b with FGF7 resulted in receptor degradation and led to increased cell proliferation. Using quantitative proteomics to explore the mechanism underlying this functional dichotomy, it was revealed that FGF10 binding resulted in the novel phosphorylation of FGFR2b at Y734, which led to the recruitment of PI3K and SH3BP4 and targeting of the receptor to recycling endosomes. Whilst the mechanism through which FGFR2b recycling promotes cell migration is not fully understood, the role of FGFR2b-PI3K-SH3BP4 complex formation in this response to FGF10 was illustrated by experiments showing that FGF10-stimulated cell migration could be inhibited by depletion of SH3BP4 or expression of a FGFR2b-Y734F mutant (Francavilla et al., 2013).

## FGF10 in Prostate Cancer

*In vivo* models have shown that elevated paracrine FGF10 stimulation of mouse prostate epithelial cells led to the development of adenocarcinoma, predominantly via activation of epithelial FGFR1. These lesions showed heightened levels of androgen receptor (AR), caused most likely by post-translational modifications augmenting AR stability. Following host castration, a subset of FGF10-induced prostate adenocarcinoma cells showed continued survival and proliferation, suggesting that paracrine FGF10 stimulation may contribute to the development of androgen independence in this murine model of prostate cancer (Memarzadeh et al., 2007). Later work using testicular feminized mice revealed that FGF10-induced prostate neoplasia is dependent on the expression of functional AR (Memarzadeh et al., 2011), demonstrating a role for FGF10-AR cross-talk in early prostate tumourigenesis.

**FIGURE 2:**
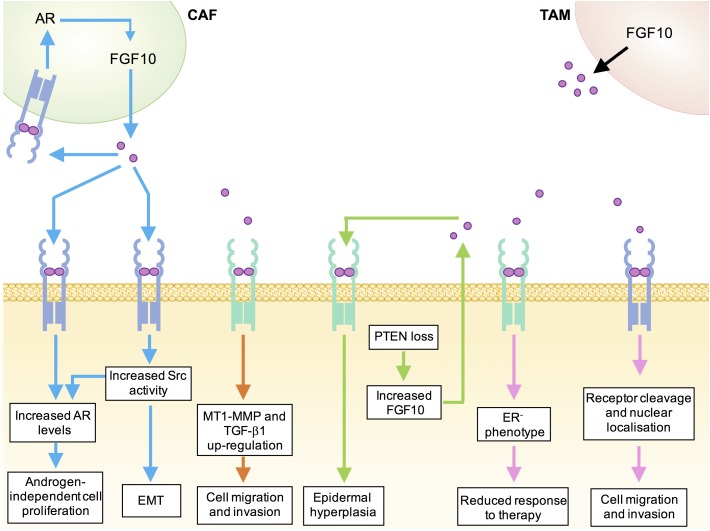
Mechanisms underlying tumor-promoting functions of FGF10 in human cancers. Blue, prostate cancer; orange, pancreatic ductal adenocarcinoma; green, cutaneous squamous cell carcinoma; pink, breast cancer; black, lung cancer; CAF, cancer-associated fibroblast; TAM, tumor-associated macrophage.

AR expression has also been detected in prostate cancer-associated fibroblasts (CAFs) *in vitro* and siRNA-mediated depletion of AR from these stromal cells resulted in a decrease in FGF10 expression. Thus, there may exist a positive feedback loop whereby increased stromal cell AR levels promote the expression of FGF10, which then acts in a paracrine manner to elevate AR levels in prostate epithelial cells and potentially also in an autocrine manner to further elevate levels of AR in the stroma (Figure 2; Yu et al., 2013).

In a model of FGF10-induced prostate tumourigenesis, enhanced phosphorylation of Src-family kinases (SFKs) was detected in the adenocarcinoma lesions. Prostate epithelium from Src^−/−^Fyn^+/−^ mice showed normal histology following exposure to elevated paracrine FGF10, suggesting that the tumourigenic effects of FGF10 on prostate epithelial cells are in part mediated by epithelial Src and Fyn kinases. Importantly, this work also established a link between FGF10, SFK signaling and AR levels by demonstrating that FGF10-stimulated Src^−/−^Fyn^+/−^ xenografts showed downregulation of AR relative to FGF10-stimulated wild type xenografts (Cai et al., 2011). Recent *in vivo* studies have provided further evidence for a role of a FGF10/FGFR/Src signaling axis in prostate cancer. Ectopic expression of FGFR1, FGFR2, or Src in mouse prostate epithelial cells growing in a normal microenvironment was not sufficient to induce prostate tumourigenesis *in vivo.* However, paracrine FGF10 was found to synergize with FGFR1/2 over-expression to induce epithelial-mesenchymal transition and FGF10 synergized with Src overexpression to induce high-grade epithelial tumors. Importantly, inhibition of Src signaling, either by pharmacological Src kinase inhibitors or by deletion of the Src myristoylation site, inhibited paracrine FGF10-induced prostate tumourigenesis (Li et al., 2018b).

Whilst these studies in mouse models of prostate cancer have revealed potential novel roles for FGF10 in androgen signaling, it is important to note that elevated FGF10 has not been detected in human prostate cancer tissue (Abate-Shen and Shen, 2007; Eiro et al., 2017) and levels of FGF10 in the normal adult prostate are extremely low, compared to those of FGF7 (Ropiquet et al., 2000). Transfection of malignant prostate tumor cells with FGFR2b cDNA has been shown to reduce the growth rate of the derived tumors (Feng et al., 1997) and restoration of FGFR2b expression in castration-resistant prostate cancer cells increased sensitivity to chemotherapeutic agents (Shoji et al., 2014). Further investigation is therefore required to fully elucidate the complex roles of FGF10-FGFR2b signaling in prostate tumourigenesis.

## FGF10 in Pancreatic Cancer

Whilst FGF10 expression is not detected in the normal adult human pancreas (Ishiwata et al., 1998), FGF10 is required for the proliferation of pancreatic epithelial progenitor cells and FGF10^−/−^ mouse embryos show pancreatic hypoplasia, arrested pancreatic epithelial branching and an absence of islet cells (Bhushan et al., 2001). Expression of FGFR2 and FGFR1 are up-regulated in approximately 25% of pancreatic adenocarcinoma (PDAC) cases and elevated stromal FGF10 expression coupled with high cancer cell FGFR2b expression has been correlated with poor prognosis (Cerami et al., 2012; Gao et al., 2013; Bailey et al., 2016). *In vitro* studies using FGFR2b-expressing pancreatic cancer cell lines revealed that FGF10 stimulation promoted cancer cell migration and invasion through the up-regulation of MT1-MMP and TGF-β1 (Figure 2; Nomura et al., 2008).

More recent work aiming to identify diagnostic and predictive markers for pancreatic cancer found that FGF10 levels were elevated in the sera of untreated patients with PDAC compared to healthy controls and found that in combination with a panel of 4 other cytokine markers, FGF10 could be used as a diagnostic biomarker for PDAC (Torres et al., 2014).

## FGF10 in Stomach Cancer

During development, FGF10 signaling plays a key role in stomach morphogenesis, through regulating gastric gland formation and maintaining an epithelial progenitor cell niche (Nyeng et al., 2007). In a cohort of 961 gastric cancers from the United Kingdom, Korea and China, *FGFR2* amplification was detected in 4.2–7.4% of cases and was associated with lymph node metastasis and poor overall survival (Su et al., 2014). *FGF10* amplification has also been detected in 3% of gastric cancers (Ooi et al., 2015) and immunohistochemical analysis of 178 gastric adenocarcinoma samples revealed that FGF10 levels are correlated with poor prognosis (Sun et al., 2015). Interrogation of most recent cBioPortal data suggests this could be an underestimate, with *FGF10* amplifications reported in 5.7% of stomach adenocarcinoma cases (Cerami et al., 2012; Gao et al., 2013).

## FGF10 in Skin Cancer

Whilst local elevation of FGF22, FGF7, and FGF10 are required for efficient healing of skin lesions (Braun et al., 2004), sustained elevation of FGF10 has also been implicated in cutaneous squamous cell carcinoma (SCC). Epidermal deletion of the tumor suppressor *Pten* produces a model of cutaneous squamous cell carcinoma in mice (Suzuki et al., 2003). The epidermis of these mice showed elevated keratinocyte expression of FGF10, with no change in levels of FGF7, FGF2, and FGF1. This increase in FGF10 was not accompanied by any *Fgf10* transcriptional changes and was found to be dependent on the elevated mTORC1 signaling resulting from loss of the negative regulator, PTEN. The specific contribution of FGF10 to carcinogenesis was demonstrated by induction of constitutive epidermal FGF10 expression, which produced epidermal hyperplasia and spontaneous papillomas in all mice by 3 weeks of age. Crucially, it was shown that genetic ablation of *Fgfr2* prevented hyperplasia in PTEN-deficient epidermis, suggesting that epidermal tumourigenesis induced by PTEN loss is mediated by an up-regulation of FGF10-FGFR2 autocrine signaling. Low levels of PTEN accompanied by elevated FGF10 have been observed in a panel of clinical SCC samples, highlighting the potential importance of this mechanism in the human disease (Hertzler-Schaefer et al., 2014).

In contrast to these data implicating FGF10 in skin carcinogenesis, previous work has suggested that FGF10-FGFR2b signaling may perform tumor-suppressive functions in the skin. Mice lacking epidermal Fgfr2b showed increased sensitivity to chemical carcinogens and 10% of animals surviving into adulthood developed spontaneous papillomas. Epidermal *Fgfr2b* ablation induced several changes in gene expression in the skin, including downregulation of Serpin a3b, a potential tumor suppressor (Grose et al., 2007).

## FGF10 in Lung Cancer

Whilst FGF10 is crucial for branching morphogenesis in the developing lung (Sekine et al., 1999), induction of FGF10 over-expression in the respiratory epithelial cells of adult mice has been shown to cause multifocal pulmonary adenomas (Clark et al., 2001). A recent study designed to identify genomic variations in cell-free DNA in small cell lung cancer patients identified *FGF10* amplification in 37.5% and of patients tested and *FGFR1* amplification in 25% of cases (Du et al., 2018). Analysis of 1144 lung cancer tumors comprising lung adenocarcinomas and lung squamous cell carcinomas revealed *FGF10* amplifications in 8.7% and FGFR1 amplifications in 9.3% of cases (Campbell J. D. et al., 2016).

The role of FGF10 in lung cancer initiation and progression remains poorly understood. However, emerging evidence suggests that FGF10 secreted from tumor-associated macrophages may play a role in promoting lung tumourigenesis (Figure 2). Tumour-associated macrophages (TAMs) are macrophages that have been co-opted by the tumor microenvironment to promote the growth and invasion of cancer cells (Noy and Pollard, 2014). Recent work showed that induction of FGF9 overexpression in the lungs of transgenic mice resulted in the development of adenocarcinoma-like nodules that are infiltrated with an immune response consisting mostly of macrophages. Expression analysis revealed that the TAMs from these transgenic mice expressed significantly higher levels of FGF2, FGF10, and FGFR2 than macrophages from wild-type mice. It has been suggested that activation of an FGF10-FGFR2 pathway may underlie the transition to FGF9-independent tumor growth, which has been observed in previous studies using this lung cancer model (Hegab et al., 2018). Expression profiling of TAMs in other pre-clinical cancer models will reveal the clinical significance of these early findings.

## Therapeutic Approaches Targeting FGF10-FGFR2 Signaling

In light of mounting evidence supporting a role for aberrant FGF10-FGFR2b signaling in tumourigenesis, FGFR2b has become an attractive therapeutic target. Whilst a number of pan-FGFR inhibitors have entered the clinic (Clayton et al., 2017), the development FGFR2-specific inhibitors is hindered by the structural similarity of the kinase domains of FGFR1-3 (Belov and Mohammadi, 2013). For this reason, ATP-mimetic inhibitors of the FGFR2b isoform are currently unavailable. However, the development of monocloncal antibodies targeting FGFR2b may provide an opportunity to target this isoform specifically. Bemarituzumab (2018) (FPA144) is an anti-FGFR2b humanized monoclonal antibody currently in Phase I clinical trials as a monotherapy for FGFR2b-amplified gastric cancers. Bemarituzumab prevents the binding of FGF10, FGF7 and FGF22 to the FGFR2b and is reported to also promote antibody-dependent cell-mediated cytotoxicity through the recruitment of natural killer cells to the tumor (*Bemarituzumab (FPA144) | Gastric Cancer | Five Prime Therapeutics*).

FGF10 signal transduction requires recruitment of the myristoylated scaffold protein FRS2α to the activated receptor, and recent pre-clinical work has highlighted the potential of targeting FRS2α myristoylation in paracrine FGF10-induced tumourigenesis. Primary mouse prostate cells transduced with FRS2α(wt) or FRS2α(G2A), a mutant that cannot be myristolyated, were mixed with mouse urogenital sinus mesenchyme (UGSM) cells expressing either FGF10 or GFP as a control. The cells were implanted sub-renally into SCID mice. Xenografts derived from FRS2α(wt) prostate cells mixed with FGF10-UGSM showed adenocarcinoma, whilst xenografts of FRS2α(G2A) cells with FGF10-UGSM cells showed normal prostate tubules. *In vitro* work showed that FRS2α myristolylation can be targeted pharmacologically by treating with the myristoyl-coA analog B13 (Li et al., 2018a).

Recent evidence has demonstrated that a FGF10/FGFR/Src signaling axis may contribute to prostate tumourigenesis via a mechanism that is dependent on Src activity. Inhibition of Src myristoylation, and therefore membrane localization, has been suggested as a viable therapeutic strategy for these prostate cancer subtypes. *In vitro* studies showed that loss of Src myristoylaton had a significant inhibitory effect on FGF10-induced oncogenic signaling in comparison with a kinase-dead Src mutant (Li et al., 2018b). These data have prompted efforts to develop an N-myristoyltransferase inhibitor as a means to therapeutically target the FGF10/FGFR/Src signaling axis in cancer (French et al., 2004; Thinon et al., 2014; Kim et al., 2017).

## Conclusion and Future Perspectives

The receptors for FGF10, FGFR2, and FGFR1 have been implicated in several human cancers, including pancreatic ductal adenocarcinoma and gastric cancer. However, since FGFR2 and FGFR1 are activated by a number of FGF family members, it has been difficult to attribute the tumor-promoting effects of these receptors to binding of a specific ligand. Recent data have begun to shed light on the role of FGF10 in these cancers, demonstrating that paracrine FGF10 synergizes with FGFR1/2 over-expression to induce epithelial-mesenchymal transition in a pre-clinical model of prostate cancer. The development of isoform-selective pharmacological tools will clarify the role of FGF10-FGFR2b/1b signaling in different cancer types and will allow the potential of FGF10 as a therapeutic target to be explored.

## Author Contributions

Both authors listed have made a substantial, direct and intellectual contribution to the work, and approved it for publication.

## Conflict of Interest Statement

The authors declare that the research was conducted in the absence of any commercial or financial relationships that could be construed as a potential conflict of interest.
